# Novel prognostic marker PRMT1 regulates cell growth via downregulation of CDKN1A in HCC

**DOI:** 10.18632/oncotarget.23296

**Published:** 2017-12-14

**Authors:** Jea-Woon Ryu, Seon-Kyu Kim, Mi-Young Son, Su-Jin Jeon, Jung-Hwa Oh, Jung Hwa Lim, Sunwha Cho, Cho-Rok Jung, Ryuji Hamamoto, Dae-Soo Kim, Hyun-Soo Cho

**Affiliations:** ^1^ Korea Research Institute of Bioscience and Biotechnology, Daejeon, Republic of Korea; ^2^ Department of Functional Genomics, Korea University of Science and Technology, Daejeon, Republic of Korea; ^3^ Korea Institute of Toxicology(KIT), Daejeon, Republic of Korea; ^4^ Division of Molecular Modification and Cancer Biology, National Cancer Center, Tokyo, Japan

**Keywords:** HCC, PRMT1, prognostic marker

## Abstract

Hepatocellular carcinoma (HCC) is a major type of liver cancer caused by the hepatitis B and C viruses, alcohol and exposure to aflatoxin. For HCC treatment, anticancer drugs have been widely used, but drug resistance in advanced HCC is an important problem, resulting in a continuous need for novel therapeutic targets. Therefore, in this study, we established a screening pipeline based on RNA-seq to screen novel therapeutic/prognostic targets in HCC and identified PRMT1 (Protein Arginine Methyltransferase 1). In the prognostic analysis, the overexpression of PRMT1 was clearly associated with poor prognosis in a number of HCC patient cohorts. Moreover, after PRMT1 knockdown, HCC cell lines exhibited cell growth and spheroid formation suppression, an increase in Sub-G1 cells by FACS analysis, and enrichment of the cell cycle pathway via functional enrichment analysis. With these results, we demonstrated that PRMT1 could be a novel prognostic marker and therapeutic target for HCC therapy.

## INTRODUCTION

Liver cancer is the second leading cause of death in cancer patients, and hepatocellular carcinoma (HCC) is the most common form of primary liver cancer. Chronic hepatitis B and C virus (HBV and HCV, respectively) infections are important causes of HCC; alcohol, exposure to aflatoxin and diabetes are other factors in HCC pathogenesis [1, 2]. Liver transplantation is the most efficient therapy to cure HCC. Chemotherapy and surgical removal are also recognized as alternative methods for HCC treatment. For example, sorafenib is a multi-kinase inhibitor for reducing HCC proliferation and angiogenesis via inhibiting Raf-1, B-Raf, VEGFRs and PDGFR [3]. However, sorafenib resistance in advanced HCC is considered a global issue. Thus, the development of novel therapeutic targets is needed [4].

HCC progression involves several deregulated signaling pathways. Among them, epigenetic regulation involving DNA methylation, histone modification (methylation/acetylation) and non-coding RNAs have been studied for drug development for HCC treatment [5–7]. The histone methylation status is highly correlated with the clinicopathological implications of HCC, and high levels of histone H3 lysine 4 trimethylation (H3K4me3) and H3K27me3 are correlated with poor prognosis in HCC [8, 9]. Several histone methyltransferases/demethylases are associated with the regulation of histone methylation in HCC [5]. EZH2, which trimethylates H3K27 for transcriptional silencing, is overexpressed and associated with HCC progression and aggressive behavior [10, 11]. The histone demethylase KDM1A is also highly expressed and is an independent predictor of worse prognoses in the 5-year overall survival of HCC [12]. However, other histone methyltransferases/demethylases have not been well-studied with respect to hepatocellular malignancy or as prognostic markers in HCC.

PRMT1 is a histone arginine methyltransferase that mainly catalyzes H3R4me and me2 to activate gene expression [13, 14]. Several papers have reported that PRMT1 expression is elevated in various types of cancer, including lung cancer, bladder cancer, breast cancer and acute myeloid leukemia, and that PRMT1 knockdown induces the suppression of cancer cell growth and metastasis [14–16]. In HCC, PRMT1 expression is targeted by miR-503, which is related to HCC metastasis regulation. However, the functions and prognostic features of PRMT1 are not yet fully understood [17]. Therefore, in this study, we identified PRMT1 overexpression using 50 normal liver and 371 HCC samples in TCGA and revealed its prognostic value in HCC using multiple patient cohorts. Thus, our findings indicate that PRMT1 may be a novel prognostic marker for HCC and that targeting PRMT1-mediated proliferation may be an effective HCC therapy.

## RESULTS

### Outline of experimental steps for screening for novel prognostic markers in HCC

**Figure 1:**
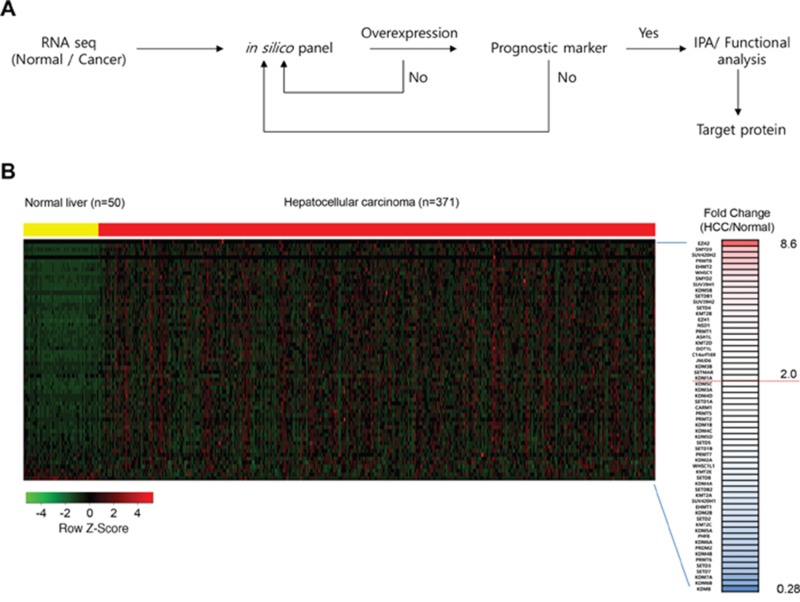
**(A)** A workflow for screening the prognostic/therapeutic marker in HCC. **(B)** A heat map of gene expression-related histone methylation/demethylation in the *in silico* histone methyltransferases/demethylases panel sorted by fold change of HCC/normal FPKM value. In the heat map, yellow indicates normal liver samples while red indicates HCC samples in TCGA. The right hand column shows gene name and fold change. The threshold is set at 2.0-fold change.

Figure 1A shows a flowchart with the following steps for constructing a screening system to identify novel prognostic markers in HCC: 1) Collect RNA-seq data (Normal/Cancer) TCGA portal; 2) Analyze expression levels using an *in silico* panel including histone methyltransferases/demethylases; 3) Test the possibility of a prognostic marker by Kaplan-Meier plot and log-rank test (*P* < 0.05); and 4) Analyze the function/pathway. Using this screening pipeline, we identified PRMT1 as a novel prognostic marker in HCC and demonstrated it as a therapeutic target for HCC treatment.

### Overexpression of histone methyltransferases and demethylases in HCC

**Figure 2:**
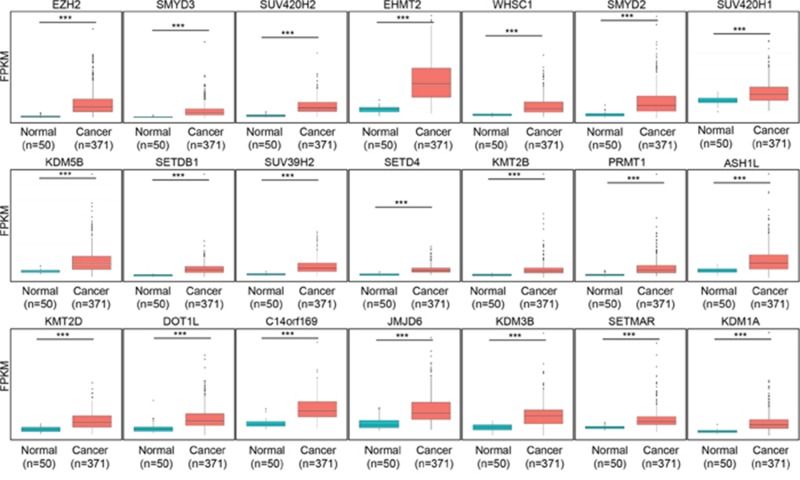
Box plots of the expression of 21 genes were grouped into normal and tumor sets in TCGA liver data set The p values were calculated using Student’s t-test (^***^p<0.001).

From the TCGA data portal, we obtained RNA-seq results from 50 normal livers and 371 HCC samples to assess HCC-related histone methyltransferases and demethylases. We used these data to construct an *in silico* gene panel with 60 histone methyltransferases/demethylases (Figure 1B) for the analysis. We observed various histone methyltransferase/demethylase expression patterns in HCC compared with normal liver tissues (Figure 1B). Specifically, the fold changes (FCs) ranged from 0.28 (KDM8) to 8.6 (EZH2), and 21 histone methyltransferases and demethylases were more than 2 FC overexpressed in HCC compared with normal livers (Figure 2). Of note, EZH2 (8.6 FC) and KDM1A (2 FC) are involved in tumor proliferation and metastasis and are recognized as prognostic/diagnostic biomarkers for HCC, implying that they are therapeutic targets for HCC treatments [10, 18]. Various studies have also reported the overexpression of histone methyltransferases/demethylases in HCC, such as SMYD2/3 [19], SUV39H1/2, G9a and Dot1L [20]. However, analyses for prognostic markers of HCC have been insufficient for these histone methyltransferases/demethylases. To elucidate whether these enzymes may be prognostic biomarkers in HCC, we examined the prognostic value of 20 histone methyltransferases and cancer outcomes using the gene expression data from the NCI patient cohort and selected *PRMT1*, which was most strongly associated with HCC patient overall survival (Supplementary Table 2 and Supplementary Figure 1).

### Predictive value of *PRMT1* in HCC patient prognosis

**Figure 3:**
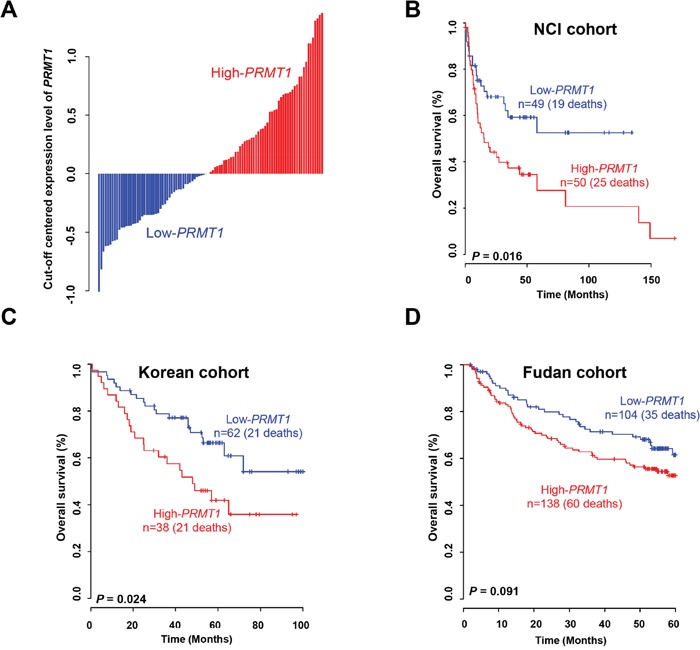
Expression of *PRMT1* and prognosis of liver cancer **(A)** Expression of *PRMT1* in HCC patients from the NCI. Each cutoff value of *PRMT1* expression was calculated by ROC analysis in each patient cohort. **(B, C, D)** Kaplan-Meier curves of overall survival in the (B) NCI, (C) Korean, and (D) Fudan cohorts.

To determine whether *PRMT1* has a prognostic value in HCC, we analyzed gene expression data from 441 samples obtained from three independent HCC patient cohorts. We divided the patients from the NCI cohort into two groups using a *PRMT1* expression threshold obtained from Receiver operating characteristic (ROC) analysis, as the frequency of overall survival was significantly higher in the high-*PRMT1* group compared with the low-*PRMT1* group (log-rank test, *P* = 0.016; Figure 3A and 3B). By applying the same procedure to the Korean cohort, a consistent statistical significance for the prediction of overall survival was also obtained (log-rank test, *P* = 0.024; Figure 3C). When estimating *PRMT1* expression in the Fudan cohort, however, we did not find a statistical significance, but instead observed a trend for classifying high-risk HCC patients by *PRMT1* expression (Figure 3D), indicating the limitations of using a single gene as a diagnostic tool.

### The suppression of HCC growth by the knocking down PRMT1 expression in HCC cell lines

PRMT1 is a protein arginine methyltransferase that catalyzes the methylation of the third arginine in histone H4 after experimental validation. In previous reports, PRMT1 was significantly elevated in several types of cancer and played an important role in cancer progression. PRMT1 expression is also associated with poor prognosis in breast and gastric cancers [16, 21], though no study has fully addressed the clinical relevance and function of PRMT1 in HCC.

**Figure 4:**
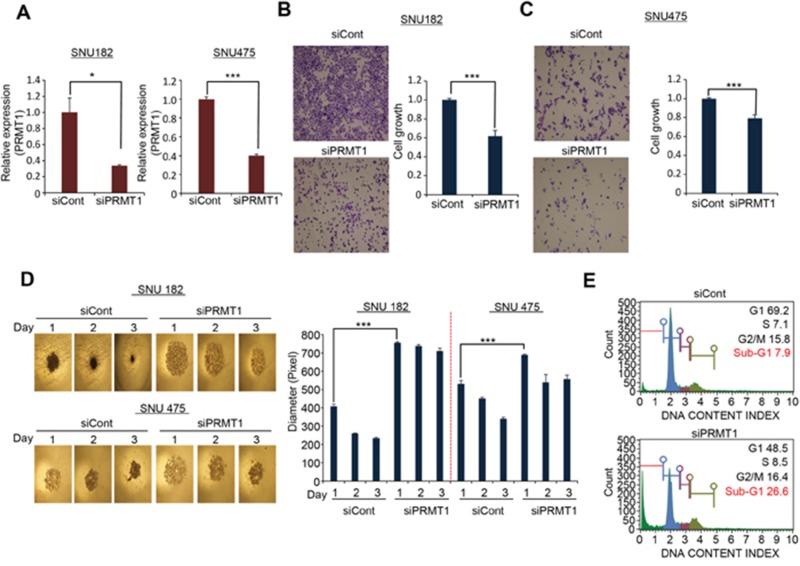
PRMT1 regulated cell growth and spheroid formation in HCC **(A)** Knockdown of PRMT1 with siRNA. qPCR analysis after treatment with siPRMT1 and siCont as a negative control for 72 h. The p values were calculated using Student’s t-test (^***^p<0.001, ^*^p<0.05). **(B** and **C)** Colony formation analysis. After knockdown of PRMT1 for 72 h, cell fixation with 100% ethanol and cell staining with crystal violet was performed (left), and the quantification of cell number was measured with a microplate reader after disassociating the colony with extraction solution (0.9% trisodium citrate dihydrate, 0.02 ml/l HCl, 50% ethanol). **(D)** Spheroid formation. Cells with siPRMT and siCont were loaded onto spheroid plates and incubated for 72 h. The cells were photographed under a microscope each day (left), and the size of spheroid was measured by image J program (right) (^***^p<0.001). **(E)** Cell cycle analysis. After knockdown of PRMT1, cell cycle analysis was performed with MUSE after staining with a cell cycle staining solution.

In HCC patients (n=371), PRMT1 expression levels gradually increased according to the HCC stages and T/N factor (data not shown), implying that PRMT1 expression may be associated with HCC malignancy and proliferation. To investigate the role of PRMT1 in HCC growth, we performed growth analysis after knocking down PRMT1 expression in HCC cell lines. qRT-PCR analysis showed that PRMT1 was clearly suppressed after treatment with siPRMT1 compared with siCont in the SNU182 and 475 cell lines (Figure 4A). In a colony formation assay, the number of HCC cells was significantly reduced after treatment with PRMT1 siRNAs (Figure 4B and 4C). The use of 3D culture models more closely reflects the tumor compared to 2D culture [22, 23]. Thus, we performed 3D culture with HCC cell lines using spheroid microplates, which are characterized by ultralow attachment. After PRMT1 knockdown, the formation of spheroids was reduced compared with siCont-treated SNU182 and 475 cells (Figure 4D). FACS analysis showed an induction of cells in Sub-G1 after PRMT1 knockdown (Figure 4E). Taken together, these results suggest that PRMT1 plays an important role in growth and spheroid formation in HCC cell lines.

### Prognostic relevance of *PRMT1* and its associated genes

**Figure 5:**
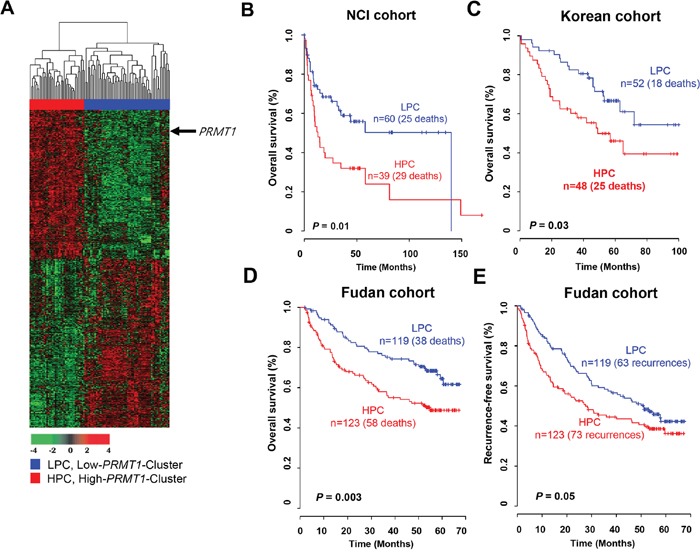
Gene expression pattern of the *PRMT1* signature and prognosis of two clusters in the NCI, Korean and Fudan cohorts **(A)** Gene expression patterns of *PRMT1* and its associated genes in the NCI cohort (n = 99). A total of 1,690 genes with expression patterns that highly correlated with *PRMT1* were selected for a cluster analysis (Pearson correlation test, |*r*| > 0.4 and *P* < 0.001). The patients were divided into two groups: a low-*PRMT1* cluster (LPC) and high-*PRMT1* cluster (HPC). **(B-D)** Kaplan-Meier plot depicting overall survival in the NCI cohort. The survival rate of the HPC patients was significantly increased compared with LPC patients (*P* = 0.01 by log-rank test). Overall survival rates of the two patient groups classified by the *PRMT1* signature were validated (C) in the Korean cohort (n = 100) and (D) in the Fudan cohort (n = 242). **(E)** The *PRMT1* signature also showed a predictive value for recurrence-free survival in the Fudan cohort.

We sought to identify a gene set that directly correlated with *PRMT1* expression to verify its prognostic relevance in HCC and explore biological interactions between *PRMT1* and its associated genes. A total of 1,690 genes correlating with *PRMT1* expression were selected from the NCI cohort (Pearson correlation test, |*r*| > 0.4 and *P* < 0.001). Based on hierarchical clustering analysis of the expression patterns of these genes, we divided the HCC samples into the following two groups: (1) the high-*PRMT1* cluster (HPC) and (2) the low-*PRMT1* cluster (LPC) (Figure 5A). The overall survival rate of the HPC patients was significantly lower than the LPC patients (log-rank test, *P* = 0.01; Figure 5B). To validate this finding in the NCI cohort, we also used gene expression data from an independent cohort of Korean patients with HCC. Using the same procedure employed with the NCI cohort, the patients in the Korean cohort were divided into two groups (HPC and LPC) by hierarchical cluster analysis using 1,672 genes that overlapped with the 1,690 genes derived from the NCI cohort, and the overall survival of each group was estimated. Kaplan-Meier analysis revealed that the *PRMT1* gene set was a significant predictor of HCC overall survival in the Korean cohort (*P*=0.03 by log-rank test; Figure 5C). When applying the same clustering algorithms and Kaplan-Meier analyses to the Fudan cohort with 1,022 overlapping genes, the survival rate of the high-risk patients was significantly lower than the low-risk patients (log-rank test, *P* = 0.003; Figure 5D). Moreover, the recurrence rate of the HPC subgroup classified by *PRMT1* and its associated genes was significantly higher than the LPC subgroup (log-rank test, *P* = 0.05; Figure 5E).

To determine the independent utility of the newly identified signature based on *PRMT1* expression, we combined the clinical data from two test cohorts and applied Cox regression analyses to our signature and known clinicopathological risk factors. In the univariate analysis, significant prognostic indicators of HCC overall survival included the *PRMT1* signature, alpha-fetoprotein (AFP), tumor size, and Barcelona Clinic Liver Cancer (BCLC) stage (Supplementary Table 3). When the multivariate test was performed on the pooled cohort, the *PRMT1* signature still retained its statistical significance for overall survival of HCC even after applying a variable selection procedure (HR = 1.612, 95% CI = 1.127 - 2.306, *P* = 0.009; Supplementary Table 3), demonstrating prognostic relevance of the *PRMT1* signature as an independent risk factor for HCC.

### Active regulators in the prognostic gene set associated with *PRMT1*

**Figure 6:**
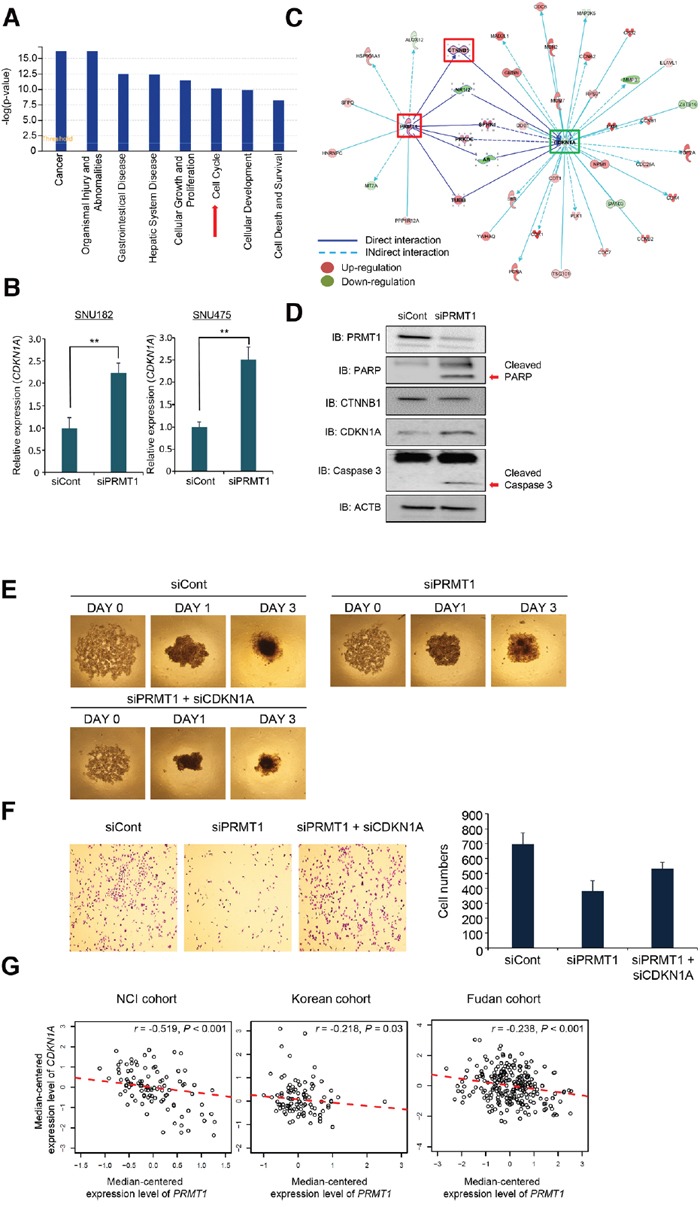
Reverse-correlation between *PRMT1* and CDKN1A in HCC clinical cohorts **(A)** Canonical pathway analysis. Histograms represent the canonical pathways. **(B)** Upregulation of CDKN1A by knockdown of PRMT1. qPCR analysis after treatment with siPRMT1 and siCont as a negative control for 72 h. The p values were calculated using the Student’s t-test (^**^p<0.01). **(C)** Gene-to-gene networks of *PRMT1* and *CDKN1A* associated with hepatocellular carcinoma (HCC) prognosis. Up- and downregulated genes in the high-*PRMT1* cluster (HPC) group are indicated in red and green, respectively. The intensity of the color is indicative of the degree of over- or under-expression. Each line and arrow represents functional and physical interactions between the genes and the direction of regulation reported in the literature. **(D)** Western blot analysis of PRMT1, PARP1, Caspase 3, CTNNB1 and CDKN1A. ACTB was used as an internal control. **(E)** Spheroid formation. Cells treated with siPRMT, siCont and siPRMT1/siCDKN1A were loaded onto spheroid plates and incubated for 72 h. The cells were photographed under a microscope each day. **(F)** Colony formation analysis. After treatment with siPRMT, siCont and siPRMT1/siCDKN1A for 72 h, the cells were fixed with 100% ethanol, stained with crystal violet (left), and the cell number was determined with the ImageJ program (right). **(G)** Scatter plots of *PRMT1* and *CDKN1A* in the NCI, Korean and Fudan cohorts. Each red dotted line indicates a linear regression line of the expression of *PRMT1* and *CDKN1A*. *P* values and correlation coefficients (*r*) between two genes were obtained by the Pearson correlation method.

To explore the biological characteristics and interactions among *PRMT1*-associated genes, a gene set enrichment test was performed using the IPA software on the 1,690 genes. The analysis showed that genes involved in cancer, cell growth and proliferation, and tissue development were significantly enriched. Among the genes associated with *PRMT1*, a significant number were identified as being involved in the cell cycle, indicating that altered biological processes associated with the cell cycle might be markedly responsible for poor prognosis in HCC (Figure 6A). Among PRMT1-associated genes, we performed a candidate approach to identify cell cycle-related genes and selected *CDKN1A*. CDKN1A, which is a cyclin dependent kinase inhibitor, drives cell cycle arrest in response to various environmental stimuli [24]. PRMT1 knockdown induced the upregulation of *CDKN1A* transcription compared with siCont-treated HCC cell lines (Figure 6B). In an analysis of *PRMT1* and *CDKN1A* gene-to-gene networks, upregulated *PRMT1* enhanced *CTNNB1* expression, and upregulated *CTNNB1* induced by PRMT1 reduced *CDKN1A* expression (Figure 6C). To confirm this result at the protein level, we performed Western blot analysis after PRMT1 knockdown and found that PRMT1 knockdown downregulated CTNNB1, induced CDKN1A, and cleaved PARP1 and Caspase 3 in the SNU182 cell line, implying that downregulated CTNNB1 induced by PRMT1 promoted CDKN1A expression and cell apoptosis (Figure 6D). Additionally, to assess whether CDKN1A is a final effector protein in this pathway, rescue experiments were performed after co-transfection with siPRMT1 and siCDKN1A. In 3D culture analysis, the cells treated with siPRMT1 had difficulty forming spheroids compared with siCont-treated cells. However, co-transfection with siPRMT1 and siCDKN1A induced spheroid formation compared to siPRMT1 treatment only (Figure 6E). Additionally, in the cell growth assay, we observed the recovery of cell growth after co-treatment with both siPRMT1 and siCDKN1A (Figure 6F). Thus, we believe that CDKN1A expression may be involved downstream of PRMT1 and that regulation of CDKN1A by PRMT1 plays an important factor for tumor growth and formation in HCC.

Finally, to validate the findings from the gene network analysis in clinical samples, we examined the rate of reverse correlation using clinical cohorts. Figure 6G shows the statistically significant reverse correlations [Pearson correlation tests; r= −0.519, *P*<0.001 (NCI); r=-0.218, *P*=0.03 (Korean); r=-0.238, *P*<0.001 (Fudan)] between *PRMT1* and *CDKN1A*. These results suggest that overexpressed PRMT1 in HCC can downregulate CDKN1A expression levels and subsequently may promote the proliferation and growth of cancer cells in response to various stimuli.

## DISCUSSION

RNA-seq is a useful tool for analyzing the whole transcriptomes of cancer cells at the levels of genes and exons involving novel splicing variants [25]. In melanoma, for the first time, novel gene fusions and read-through transcripts were identified using RNA-seq to discover cancer targets [26]. In this study, we established a screening pipeline based on RNA-seq to identify overexpressed histone methyltransferases as prognostic markers in HCC. The advantages of RNA-seq include its high sensitivity and quantification of gene expression levels. In the screening pipeline, we used public RNA-seq data from 50 normal livers and 371 HCC tissues derived from the TCGA portal and analyzed the expression levels with an *in silico* panel of histone methyltransferases/demethylases (Figure 1). We selected 21 of these histone methyltransferases/demethylases with a greater than 2-fold change in expression in HCC compared with normal tissues. Among them, we assessed the possibility of these genes being prognostic biomarkers in HCC using the NCI cohort. Several prognostic systems for estimating tumor stage have been developed, including the CLIP score, Okuda staging, and BCLC. Okuda staging and BCLC are based on tumor size and serum albumin and bilirubin levels, and the CLIP score predicts HCC based on the Child-Pugh stage, AFP level and tumor morphology [27]. However, there are limits to predicting the stage of HCC patients with the currently used systems. Therefore, to better evaluate HCC patients, novel prognostic biomarkers and assessment systems have been continuously required. Here, using our screening pipeline, we identified PRMT1 as a novel prognostic biomarker for the prediction of a response to therapeutic intervention in HCC (Figure 3).

PRMT can be classified according to the position of methylation, including three types (I, II and III). Of note, type 1 PRMTs (1, 3, 4, 6 and 8) have been thought of as attractive therapeutic targets for developing specific inhibitors because of their overexpression in various types of cancers [28]. Among them, the overexpression of PRMT1 has been observed in lung, breast, bladder, colon, and prostate cancer and leukemia [29]. Here, we also demonstrated the overexpression of PRMT1 in HCC clinical samples and that the PRMT1 knockdown inhibited growth and spheroid formation in HCC cell lines, implying that PRMT1 inhibitors may be an effective therapeutic method in HCC treatment. PRMT1 has three distinct isoforms, with PRMT1 v1 being the major isoform [30]. In colon cancer, the levels of the PRMT1 v1 isoform are clearly associated with clinical and pathological variables [31]. Additionally, in breast cancer, patients with low expression of PRMT1 v1 display longer disease-free survival [16]. However, in gastric cancer, low expression of PRMT1 is associated with poor prognosis [21]. In HCC, as with colon and breast cancer, high expression of PRMT1 was significantly related to a poor prognosis, which was confirmed with the Korean cohort. Moreover, the PRMT1-associated gene set clearly reflected the poor prognosis in the NCI, Korean and Fudan cohorts. Prognostic markers can help lead to clinical treatments by identifying patients with similar cases. Moreover, prognostic markers can also help compare differences between clinical trials and patient groups in accordance with risk factors [32]. Therefore, the novel prognostic marker PRMT1 in HCC may help identify subgroups of patients and be used to design new treatments for HCC treatment.

Recently, several papers have demonstrated that RAB27B regulates cell cycle progression through the PI3K/AKT/CDKN1A pathway and by modulating cell growth in HCC cell lines, suggesting that upregulation of CDKN1A contributes to HCC cell growth. Moreover, MDIG expression levels could effect CDKN1A expression through H3K9 tri-methylation and the induction of CDKN1A expression by MDIG knockdown associated with HCC growth [33, 34]. And Wang’s group also demonstrated that overexpression of miR-95-3p regulated expression of P21 for hepatocarcinogenesis [35]. Taken together, we could suggest that the regulation of CDKN1A in HCC by several effector genes including PRMT1 can influence tumor growth.

In summary, our screening pipeline system was easily applied to identify a novel prognostic marker in several types of cancer. PRMT1 is a novel prognostic marker and a potentially therapeutic target that may play an important role in HCC treatment.

## MATERIALS AND METHODS

### Datasets of HCC patients including TCGA data

A total of four cohorts of patients with HCC including the TCGA data were used for this study. The mRNA expression (RNA-Seq) data was obtained from TCGA data portal (http://cancergenome.nih.gov) for liver related 421 samples (50 normal samples and 371 tumor samples). We downloaded RNA-Seq quantification data (HTSeq-FPKM) and calculated mean value for the expression levels of each gene across samples. These mean values represent expression each gene about normal and tumor. For verifying a prognostic value of *PRMT1*, we also obtained gene expression datasets of HCC patients from the National Cancer Institute (GSE1898, n = 99, the NCI cohort), Fudan University Liver Cancer Institute (GSE14520, n = 242, the Fudan cohort), and Seoul and Chonbuk National University of Korea (GSE16757, n = 100, the Korean cohort), which are available from the NCBI’s GEO database. The baseline characteristics of the three cohorts of HCC patients for verifying prognostic relevance of *PRMT1* were described in Supplementary Table 1.

### Cell culture

The human liver cancer cell lines SNU 182 and 475 were purchased from the Korean Cell Line Bank (Seoul, South Korea) and were cultured in RPMI supplemented with 10% fetal bovine serum (FBS) and 1% penicillin/streptomycin in a humidified atmosphere of 5% CO_2_ at 37°C according to the manufacturer’s instructions [36].

### siRNA transfection

Control, PRMT1 and CDKN1A siRNA duplexes were purchased from ST Pharm (Seoul, South Korea) and Bioneer (Seoul, South Korea). siCont (negative control siRNA) was used for the control treatment. siRNA sequences are described in Supplementary Table 4. In total, 100 nM siRNAs were transfected into cancer cell lines using RNAiMAX (Invitrogen, Carlsbad, CA) for 48 h.

### Western blot analysis

Western blotting was performed according to the manufacturer’s instructions [36]. The cells were washed once with PBS and then lysed in cold lysis buffer (50 mM Tris-HCl, pH 7.4, 150 mM NaCl, 1% Triton X-100, 0.1% SDS, 1 mM EDTA, 1 mM Na_3_VO_4_, 1 mM NaF, and 1 × protease inhibitor cocktail). Cell lysates were centrifuged at 14,000 × g for 15 min at 4°C and then boiled in 5 × sample buffer. The protein samples were subjected to Western blotting with the indicated antibodies at a 1:500 dilution ratio. The samples were stained with the anti-PRMT1 (B-2), ACTB (SC-4778) and B-catenin (SC-7963) antibodies from Santa Cruz (Santa Cruz, CA, USA) and the anti-PARP (#9542S) and Caspase 3 (#9665S) antibodies from Cell Signaling Technology (MA, USA).

### Semi-quantitative reverse transcription PCR and quantitative real-time PCR

Total RNA was isolated from the indicated cell lines using a Qiagen RNeasy Mini Kit according to the manufacturer’s instructions [37–39]. RNA aliquots of 1 μg were then reverse transcribed using the iScript™ cDNA synthesis kit (Bio-Rad, Hercules, CA), according to the standard protocols. For semi-quantitative RT-PCR, cDNA was used as a template for PCR using AccuPower® ProFi Taq PCR PreMix (Bioneer, Daejeon, South Korea). For quantitative RT-PCR, PCR reactions were performed using the CFX96 Real-Time System (Bio-Rad) following the manufacturer’s instructions. PCR primers were as follows: PRMT1 (Forward primer 5′-GGGCTACTGCCTCTTCTACGAGTC-3′, Reverse primer 5′-GTCTTTGTACTGCCGGTCCTCGTAG-3′), CDKN1A (Forward primer 5′-GGGATGTCCGT CAGAACCCA -3′, Reverse primer 5′-CACCCTCCAGTGGTGTCTCG -3′).

### Spheroid formation

To perform spheroid culture of HCC cell lines, ultralow attachment microplates were used (Corning, Cat. 4515). After knockdown of PRMT1, a concentration of 2 × 10^4^ cells was loaded onto spheroid culture plates for 3 days then observed using microscopy.

### FACS analysis

After knockdown of PRMT1 for 3 days, the collected cells were washed with PBS and fixed with 70% cold ethanol for 3 h at −20 degrees. After washing with PBS, cell cycle solution (Millipore, MCH100106) was added and the cells were analyzed using a MUSE cell analyzer.

### Statistical analysis

To classify patients into two groups by *PRMT1* expression in the HCC gene expression data, we obtained an optimal cutoff of *PRMT1* expression from ROC analysis, in which the best cutoff was determined by the expression with the highest sensitivity and specificity. The Kaplan-Meier method was used to calculate the time to death or recurrence, and differences between the times were assessed using log-rank statistics. Pearson correlation coefficients were calculated to evaluate the association between *PRMT1* and its correlated genes. A hierarchical clustering algorithm, using the centered correlation coefficient as the measure of similarity and complete linkage clustering, was applied. The prognostic association between PRMT1 signature and clinico-pathological risk factors was assessed using multivariate Cox proportional hazard models. A backward-forward step procedure (function step, R package stats) was applied to optimize the multivariate model with the most informative variables.

A gene set enrichment analysis was performed to identify the most significant gene sets associated with the disease process, molecular and cellular functions, and physiological and development conditions. The significance of over-represented gene sets was estimated by the Fisher exact test. To explore the relationships between the genes in the gene set associated with *PRMT1*, we performed an upstream regulator analysis that searched known targets of each regulator in the data set and compared their direction of change to the expected change based on previously published literature. Gene set enrichment and upstream regulator analyses were performed using the Ingenuity Pathway Analysis (IPA, Ingenuity Systems, www.ingenuity.com).

## SUPPLEMENTARY FIGURE AND TABLES


