# Supplementary material for: Novel prognostic marker PRMT1 regulates cell growth via downregulation of CDKN1A in HCC
Source: Oncotarget. 2017 Dec 14;8(70):115444–55. doi: 10.18632/oncotarget.23296 (PMC5777784; doi:10.18632/oncotarget.23296)
Supplement: Supplementary file 1 [file oncotarget-08-115444-s001.pdf]

Novel prognostic marker PRMT1 regulates cell growth via downregulation of CDKN1A in HCC

SUPPLEMENTARY MATERIALS

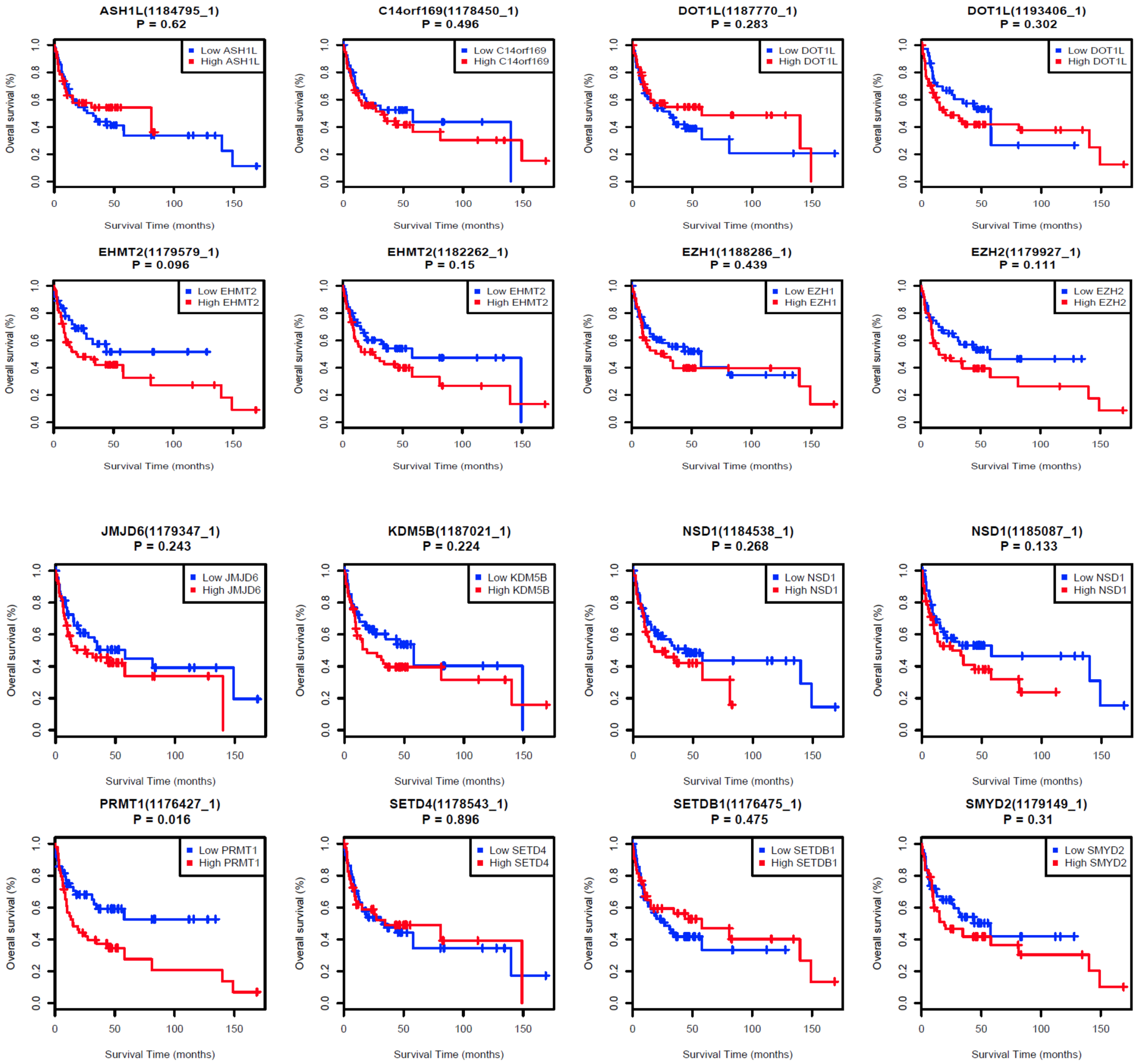

(*Continued*)

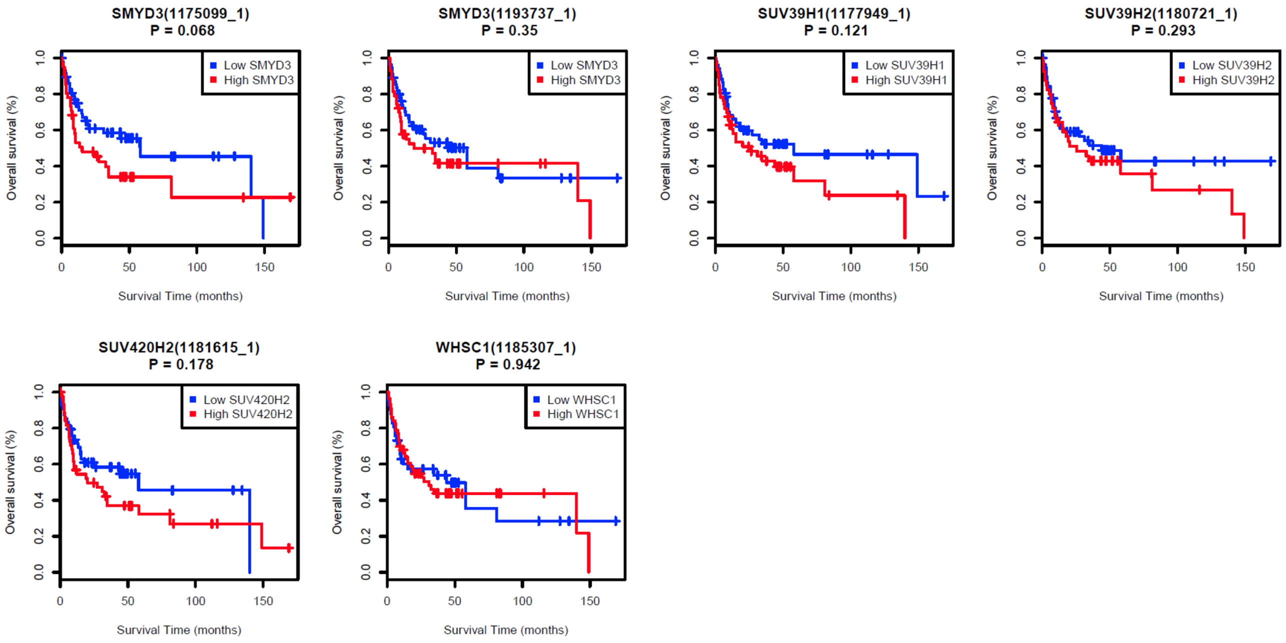

Supplementary Figure 1: Prognostic value estimation of 20 histone methyltransferases in hepatocellular carcinoma patients in the NCI cohort. Among the 20 genes, the probes corresponding to *KMT2B* and *KMT2D* did not exist in the microarray data of the NCI cohort. Therefore, remained 18 genes (22 probes) were applied to this survival analysis. Each *P*-value was obtained by a log-rank test. The number in the bracket beside the gene symbol indicates the unique probe idedntifier of the microarray data in the cohort.

Supplementary Table 1: Baseline characteristics of hepatocellular carcinoma patient cohorts

Variables	NCI cohort	Korean cohort	Fudan cohort
Patients (n)	99	100	242
Gender, n (%)			
Male	77 (77.8)	83 (83)	211 (87.2)
Female	22 (22.2)	17 (17)	31 (12.8)
Age (years)			
Median	53	55	50
Range	26-77	25-70	21-77
HPV, n (%)			
+	49 (49.5)	58 (58)	218 (90.1)
-	50 (50.5)	17 (17)	6 (2.5)
NA		25 (25)	18 (7.4)
AFP (>300 ng/mL), n (%)			
+	49 (49.5)	32 (32)	110 (45.5)
-	45 (45.4)	68 (68)	128 (52.9)
NA	5 (5.1)		4 (1.6)
AJCC stage, n (%)			
I		35 (35)	96 (39.7)
II		17 (17)	78 (32.2)
III		48 (48)	51 (21.1)
IV		0 (0)	0 (0)
NA	99 (100)		17 (7)
BCLC stage, n (%)			
O		0 (0)	20 (8.3)
A		53 (53)	152 (62.8)
B		37 (37)	24 (9.9)
C		6 (6)	29 (12)
D		4 (4)	0 (0)
NA	99 (100)		17(7)
Death, n	54	43	96
Median follow-up (month)	20.5	48.5	51.7

Abbreviations: HBV, hepatitis B virus; AFP, alpha-fetoprotein; AJCC, American Joint Committee on Cancer; BCLC, Barcelona Clinic Liver Cancer; NA, not available.

Supplementary Table 2: Prognostic values of 20 histone methyltransferases for overall survival of hepatocellular carcinoma patients in the NCI cohort

Gene symbol	Probe ID	*P*-value*
PRMT1	1176427_1	0.016
SMYD3	1175099_1	0.068
EHMT2	1179579_1	0.096
EZH2	1179927_1	0.111
SUV39H1	1177949_1	0.121
NSD1	1185087_1	0.133
EHMT2	1182262_1	0.15
SUV420H2	1181615_1	0.178
KDM5B	1187021_1	0.224
JMJD6	1179347_1	0.243
NSD1	1184538_1	0.268
DOT1L	1187770_1	0.283
SUV39H2	1180721_1	0.293
DOT1L	1193406_1	0.302
SMYD2	1179149_1	0.31
SMYD3	1193737_1	0.35
EZH1	1188286_1	0.439
SETDB1	1176475_1	0.475
C14orf169	1178450_1	0.496
ASH1L	1184795_1	0.62
SETD4	1178543_1	0.896
WHSC1	1185307_1	0.942

* *P*-values were obtained by log-rank test.

Supplementary Table 3: Univariate and multivariate Cox regression analysis of overall survival

Variable	Univariate			Multivariate		
	n	HR (95% CI)	*P*-value	*n*	HR (95% CI)	*P*-value
**Gender (Male or Female)**	342	1.336 (0.793 - 2.249)	0.276	342		
**Age (>60 years or not)**	342	0.897 (0.592 - 1.359)	0.608			
**AFP (>300 ng/mL or not)**	342	1.682 (1.204 - 2.35)	0.002			
**Tumor size (>5 cm or not)**	342	2.053 (1.471 - 2.865)	<0.001		1.966 (1.379 - 2.803)	<0.001
**BCLC stage (B/C/D or 0/A)**	342	1.816 (1.285 - 2.566)	0.001		1.46 (1.023 - 2.085)	0.037
***PRMT1* signature (LPC vs. HPC*)**	342	1.867 (1.329 - 2.621)	<0.001		1.612 (1.127 - 2.306)	0.009

* Predicted outcome in Figure 5 was used for analysis [low *PRMT1* cluster (LPC) or high *PRMT1* cluster (HPC)].
Abbreviations: HR, hazard ratio; CI, confidence interval; AFP, alpha-fetoprotein; BCLC, Barcelona Clinic Liver Cancer.

Supplementary Table 4: Sequences for siRNA

Gene name	sequence
Control siRNA	5’-AUGAACGUGAAUUGCUCAATT-3’
	5’-UUGAGCAAUUCACGUUCAUTT-3’
PRMT1 siRNA	5’-GAGUUCACACGCUGCCACATT-3’
	5’-UGUGGCAGCGUGUGAACUCTT-3’
CDKN1A siRNA	5’-CUGUACUGUUCUGUGUCUUTT-3’
	5’-AAGACACAGAACAGUACAGTT-3
